# PET-CMR in heart failure - synergistic or redundant imaging?

**DOI:** 10.1007/s10741-017-9607-6

**Published:** 2017-03-20

**Authors:** Michael A. Quail, Albert J. Sinusas

**Affiliations:** 1 Section of Cardiovascular Medicine, Department of Medicine, Yale University School of Medicine, Dana 3, P.O. Box 208017, New Haven, CT 06520-8017 USA; 2 Institute of Cardiovascular Science, University College London, London, UK; 3 Department of Radiology and Biomedical Imaging, Yale University School of Medicine, New Haven, CT USA

**Keywords:** Heart failure, PET, CMR, Cardiomyopathy, Diagnostic imaging

## Abstract

Imaging in heart failure (HF) provides data for diagnosis, prognosis and disease monitoring. Both MRI and nuclear imaging techniques have been successfully used for this purpose in HF. Positron Emission Tomography-Cardiac Magnetic Resonance (PET-CMR) is an example of a new multimodality diagnostic imaging technique with potential applications in HF. The threshold for adopting a new diagnostic tool to clinical practice must necessarily be high, lest they exacerbate costs without improving care. New modalities must demonstrate clinical superiority, or at least equivalence, combined with another important advantage, such as lower cost or improved patient safety. The purpose of this review is to outline the current status of multimodality PET-CMR with regard to HF applications, and determine whether the clinical utility of this new technology justifies the cost.

## Introduction

Heart failure (HF) is an ever-increasing public health problem, with an annual global economic burden of $108 billion per annum [1]. The magnitude of this problem mandates innovative therapeutic and diagnostic solutions. However, the threshold for adopting new diagnostic tools to clinical practice must necessarily be high, lest they exacerbate costs without improving care. New modalities must demonstrate clinical superiority, or at least equivalence, combined with another important advantage, such as lower cost or improved patient safety.

Positron Emission Tomography-Cardiac Magnetic Resonance (PET-CMR) is an example of a new multimodality diagnostic imaging technique with potential applications in heart failure. In PET-CMR, PET data is acquired either simultaneously or sequentially to CMR data using hybrid-scanner infrastructure. Such hardware has been available for approximately 5 years, however the high costs has limited utilization to a small number of early adopters; not necessarily focused on cardiac applications – therefore the literature at this time remains sparse. As an alternative to PET-CT, PET-CMR is associated with lower radiation exposure, improved cardiac and respiratory motion compensation and versatile data acquisition. However there are important concerns about capital (~$9,000,000) and operational costs (~$100,000 per month [2]), technical limitations and patient eligibility.

CMR and PET scans have been successfully used in the evaluation of patients with HF either simultaneously or separately with application of post-acquisition fusion of independently acquired scans. The purpose of this review is to outline the current status of the new hybrid PET-CMR technology with regard to HF applications – Is hybrid PET-CMR now ready for prime time in HF?

## Multimodality imaging in HF

Cardiac pump dysfunction is the final endpoint of a range of cardiac pathologies, such as ischemic heart disease, replacement fibrosis, infiltration, inflammation and myocardial iron deposition. Establishing the specific etiology of HF is important because it is associated with prognosis [3] and guides specific therapy: for example, revascularization and secondary prevention for ischemic heart disease. While imaging is generally helpful in providing a specific diagnosis, variability in diagnostic performance can often result in etiologic uncertainty. Multi-modality imaging aims to harness the different physical bases of image formation to create an image set with complementary (or even synergistic) information. The aim is to produce superior biomarkers for heart failure diagnosis, prognosis, and monitoring. For example improved statistical performance for risk estimation by using PET biomarkers with CMR cardiac or respiratory motion correction [4], or improved diagnostic categorization of patients according to differential patterns of fused PET-CMR images.

By contrast, if data from different imaging modalities provide the ‘same’ information in different image spaces, collinearity and redundancy are introduced. The success or failure of PET-CMR (and indeed all multi-modality imaging) is therefore dependent on acquiring information more valuable than the individual components.

### PET imaging

Cardiac PET is the modality of choice to measure perfusion, metabolism and regional/absolute myocardial blood flow. PET imaging is facilitated by the use of radiotracers, in which a compound or pharmaceutical is labeled with a positron emitting radionuclide. Positrons are positively charged nuclear particles with the same mass as an electron. When an emitted positron collides with an electron in adjacent tissue, two 511 keV gamma rays (photons) are emitted in opposite directions. PET detectors are designed to register only photon pairs that strike opposing detectors at the same time, termed ‘coincidence detection’. PET radionuclides generally have much shorter half-lives than those used in single photon emission CT (SPECT), and therefore are associated with less radiation exposure [5].

The most commonly utilized cardiac PET tracers include: tracers to assess myocardial blood flow (MBF) (^13^N–Ammonia, Rubidium-82 [^82^Rb], oxygen-15 labeled water [H_2_^15^O]) or glucose metabolism (^18^F–Fluorodeoxyglucose, FDG). The type of image that can be acquired is based upon the pharmacokinetics of the particular radiopharmaceutical, and in principle, any physiological or molecular process that can be targeted with a radiotracer can be imaged, given suitable uptake in the area of interest. An important area of translational imaging research is the clinical development of specific molecular probes, which can detect inflammation, thrombosis, apoptosis, necrosis, angiogenesis, fibrosis or other alterations of the extracellular matrix [6].

Measurement of absolute MBF by PET entails the intravenous injection of a MBF tracer followed by dynamic acquisition of images of the radiotracer passing through the circulatory system and evaluation of extraction and retention in the myocardium. Kinetic models and operational equations (accounting for tracer decay, partial volume, blood pool spillover) are then applied to yield regional MBFs in absolute terms, ml/g/min [7]. Calculating the ratio of peak MBF at maximal pharmacologically induced vasodilatation to resting MBF provides quantification of myocardial perfusion reserve, which provides important prognostic and severity information in the assessment of coronary stenosis and microvascular disease [8–10].

^18^F–FDG is a glucose analog that is taken up by myocytes (and other cells, including immune cells) via membrane glucose transporters (GLUT); and therefore highly sensitive to metabolically active processes. ^18^F–FDG is phosphorylated intracellularly in the glycolytic pathway by a hexokinase into ^18^F–FDG-6-phosphate. However ^18^F–FDG-6-phosphate cannot be metabolized further along this pathway and therefore accumulates within cells in direct proportion to their metabolic activity. In the myocardium, under aerobic conditions, most myocardial energy consumption is derived from oxidation of free fatty acids, followed by glucose, and therefore accumulation of ^18^F–FDG-6-phosphate is expected to be low, but increased in areas of increased metabolism, such as inflammation, or in the setting of myocardial hibernation.

### CMR imaging

CMR is an excellent non-invasive imaging modality for characterization of the heart as a complex structure and mechanical pump. Cine imaging is the reference standard for the assessment of structure and global ventricular performance through quantification of chamber volumes and function (ejection fraction (EF) and cardiac output). Combined with phase contrast imaging, valve regurgitation volume/fraction can also be reliably estimated for all four cardiac valves. Cine imaging can also be used to demonstrate regional wall motion abnormalities or LV thrombus. Newer forms of acquisition can directly assess myocardial deformation or *strain* (e.g. Tagging, Tissue phase mapping or DENSE imaging) [11, 12]. Assessment of diastolic function can also be performed with combinations of the above techniques, with characterization of atrial volume and mitral valve inflow patterns. In serial follow-up of ventricular function, CMR offers markedly superior inter-study reproducibility compared to 2D echocardiography [13].

CMR can also be used to characterize myocardial tissues based upon three properties that determine image contrast in an MR image: proton density (density of hydrogen atoms) and two magnetic relaxation parameters (T1 – longitudinal relaxation time) and T2 (Transverse relaxation time). For example, in T1-weighted images, myocardial tissue is dark while fat is bright. By contrast, T2-weighted images can be used to identify myocardial water/edema caused by inflammation or acute ischemia. Using a gadolinium-based contrast, which acts to shorten T1 in the area of distribution, can further assist tissue characterization. Gadolinium accumulates in areas of increased extracellular volume such as scar or fibrosis, resulting in hyperenhancement on inversion recovery CMR images – late gadolinium enhancement (LGE). It is possible, therefore, using a combination of acquisitions, which differ in terms of tissue contrast, to characterize normal from abnormal areas of myocardium and identify possible pathological mechanisms (e.g. fibrosis, edema, ischemia). Gadolinium CMR may also be used to quantify myocardial perfusion and perfusion reserve (in a conceptually similar way to PET) without radiation exposure using dual bolus, first pass imaging with Fermi function deconvolution methods [14, 15].

It is immediately clear that combining PET and CMR has great potential for the characterization of pathology from molecular to organ levels; this multimodality approach may benefit investigation of patients with heart failure.

## PET-CMR in heart failure

### Ischemic heart disease

#### Myocardial perfusion imaging

The estimated prevalence of coronary artery disease (CAD) in patients with HF ranges from 50 to 65% [16, 17]. Chronic left ventricular dysfunction in patients with coronary artery disease may be due to scar, ischemia, stunning and hibernation, or a mixture – some of which may be amenable to recovery following revascularization. Using a combination of perfusion and viability imaging it is possible to define the percentage of abnormal myocardium in each of these categories.

The landmark STICH (Surgical Treatment for Ischemic Heart Failure) trial showed that surgical revascularization of patients with ischemic cardiomyopathy (LVEF <35%) improves cardiovascular mortality [18]. The identification of coronary artery disease in new onset heart failure is therefore of utmost importance. Yet, it is estimated that only 17.5% of patients hospitalized for new onset heart failure undergo diagnostic workup for CAD during their admission, increasing to only 27.4% at 90 days [19]. The diagnosis of obstructive coronary artery disease by myocardial perfusion imaging (MPI) is the most frequent indication for nuclear cardiology in clinical practice and PET is the gold standard for this purpose [14, 20, 21]. Studies of MPI using hybrid PET-MRI systems are notably limited, in part because of the incompatibility with rubidium-82 generators with MRI. ^13^N–Ammonia is an alternative and MRI compatible MPI radiotracer, but requires an on-site cyclotron. The feasibility of stress MPI using ^13^N–Ammonia PET-CMR has been reported in a small number of patients and has shown good diagnostic accuracy compared to SPECT [22, 23]. Considering the challenges of performing MPI in this setting, it is likely that CMR, rather than nuclear, approaches would be preferred for PET-CMR. The most obvious solution would be the use of dobutamine/adenosine first-pass contrast-enhanced perfusion CMR. A recent meta-analysis of 19 studies and 111,636 patients with a mean follow-up of 32 months showed that patients with ischemia on a stress CMR study had an almost 8-fold increased incidence of MI and a 7-fold increased risk for cardiovascular death compared with those without ischemia [24]. A similar analysis of 12,178 patients showed that the annualized event rate after a negative CMR stress test was 1% [25]. Accurate quantitative myocardial perfusion imaging is also possible using CMR with good comparability to PET [14]. Thus, multi-parametric CMR may be the preferred option for the detection of clinically significant CAD in HF when using PET-CMR infrastructure.

#### Myocardial viability imaging

Meta-analysis data supports the view that, the greater the amount of ‘hibernating’ versus non-viable myocardium, the better the outcome with revascularization [26]. In patients with ischemic cardiomyopathy, myocardial viability imaging may be used to support a decision for revascularization eligibility. Such an assessment aims to separate myocardial dysfunction into two main categories: 1) irreversible myocardial necrosis due to prior MI with resultant scar or (2) myocardium with reversible loss of contractility as a result of chronic hypoperfusion (hibernation) or recurrent transient ischemia (repetitive stunning).

However, the role of viability imaging in this cohort remains somewhat controversial. In the PARR-2 study, patients with severe LV dysfunction and suspected CAD were randomized to FDG-PET assisted management or standard care. The study did not demonstrate a significant reduction in cardiac events for FDG PET-assisted management versus standard care. However, in those who adhered to PET recommendations and in patients without recent angiography, significant benefits were observed [27, 28]. In the viability substudy of STICH (using SPECT or dobutamine stress echo) the assessment of myocardial viability did not identify patients with a differential survival benefit from CABG, as compared with medical therapy alone [29]. These data have been criticized as only 19% of the patients in the viability study had nonviable myocardium, and the viability imaging techniques were not themselves randomized or standardized. Furthermore, PET-FDG or delayed CMR were not performed [30].

More recent data using multi-modal imaging with stress/rest, viability/perfusion imaging with PET-FDG and Rubidium-82 (identifying ischemia, scar, and hibernating myocardium) by Ling et al. [31]. do provide evidence that hibernating myocardium identified by imaging does identify those patients with ischemic cardiomyopathy who accrue a survival benefit with revascularization. In this study, the investigators found that revascularization in the setting of significant myocardial hibernation (but not scar or ischemia) improved survival, particularly when the extent of viability exceeded 10% of the LV myocardium.

**Fig. 1:**
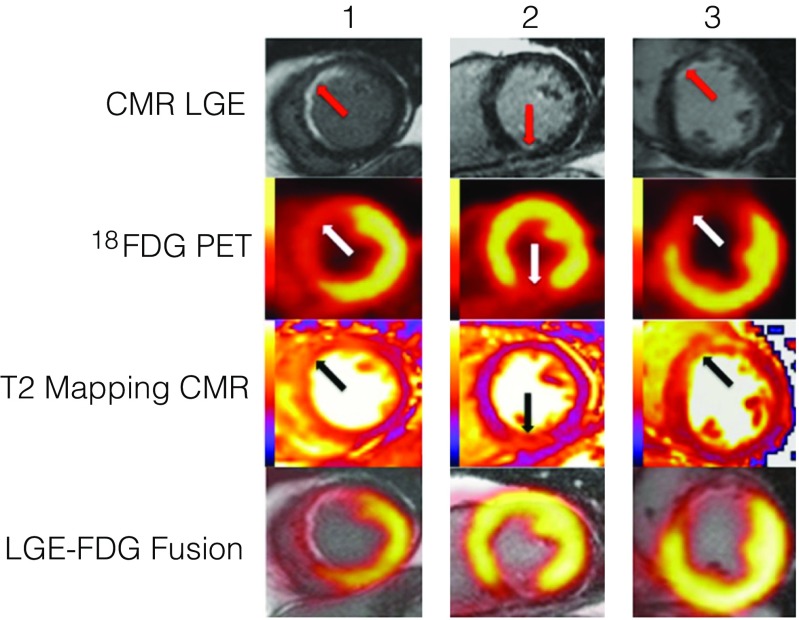
Hybrid positron emission tomography– magnetic resonance (PET-CMR) cardiac imaging of subendocardial infarction. Three study patients with subendocardial myocardial infarction (red arrows) with significant myocardial salvage. The areas of reduced ^18^F–fluorodeoxyglucose (FDG) uptake (white arrows) and the areas of edema on T2 maps (the AAR; black arrows) extended both transmurally and radially beyond the areas of late gadolinium enhancement (LGE). AAR indicates area at risk. Figure from adapted from Bullock et al. *[36]Circ CV Imaging 2016*

Both CMR and PET have been independently used to evaluate myocardial viability using LGE and ^18^F–FDG, respectively [32–35]. However, the higher spatial resolution of CMR facilitates the identification of scar more easily than PET, suggesting a benefit for hybrid imaging [33]. Several studies have investigated the role of hybrid PET-CMR for assessment of myocardial viability, albeit in acute myocardial infarction rather than ischemic cardiomyopathy. Bulluck et al. [36] used PET-CMR to predict the outcome of an ischemic injury at 12 months. In this study the salvaged ‘area at risk’ (AAR) in reperfused acute myocardial infarction (MI) was delineated using T2 mapping (Fig. 1). In patients with minimal myocardial salvage following ST elevation MI, the areas of reduced FDG uptake were confined to the areas of LGE (indicating the area irreversible myocardial necrosis). In contrast, in patients with significant myocardial salvage, the areas of reduced FDG uptake extended beyond areas of LGE. Importantly, at 12 months the study showed normalization of cardiac metabolism in the salvaged myocardium in the AAR, with only the irreversibly injured myocardium demonstrating reduced FDG uptake. The strength of this study is demonstration of the ability of PET-CMR to provide metrics relating to the extent, severity and outcome of an ischemic injury, which may be transferable to viability imaging in ischemic cardiomyopathy.

Rischpler et al. [37] used hybrid PET-CMR with the aim of comparing LGE CMR with ^18^F–FDG PET in dysfunctional myocardial segments early after acute myocardial infarction and functional recovery of these segments after 6 months. They also showed that FDG uptake was related to the AAR and exceeded the extent of LGE. This study showed that FDG uptake extent correlated with the inflammatory response as measured by blood inflammatory leukocytosis and that uptake in the infarcted myocardium was associated with LV function independent of infarct size.

### Coronary imaging

Coronary PET-CMR imaging using ^18^F–FDG and ^18^F–sodium fluoride radiotracers in combination have been used recently to image coronary artery inflammation and microcalcification, respectively. This very new approach, has the potential to be used to identify patients with increased or active atherosclerotic disease activity that may benefit from aggressive risk factor modification [38].

### Sarcoid

Sarcoidosis is a systemic granulomatous disease in which inflammatory granulomas result in marked local inflammation and post-inflammatory scar. Cardiac granulomas produce conduction defects, heart block and heart failure [39]. The onset of cardiac involvement heralds a markedly worse prognosis for affected patients [40]. Early diagnosis and intervention with disease modifying therapy is therefore warranted. Historically Gallium-67 scintigraphy has been used for the diagnosis of cardiac sarcoid, but it has a low sensitivity owing to low image resolution, and has begun to be replaced by other imaging modalities such as CMR or PET. CMR has the potential to demonstrate myocardial edema using T2-weighted imaging, and also delineate scarring with LGE imaging, which has prognostic significance [41]. Importantly, inflammation is an important component of the disease process, and ^18^F–FDG PET may provide unique data on disease activity. Ishimaru et al. [42] showed abnormalities in PET imaging that were entirely absent on traditional Gallium scans. The complementary nature of CMR and PET imaging modalities has been investigated by Ohira et al. [43] who studied 21 patients with cardiac sarcoid using serial imaging techniques performed within 4 weeks. In this cohort of patients, the authors found focal ^18^F–FDG uptake in 15 patients (71%), and high signal intensity (T2-weighted imaging) or LGE on CMR in 9 patients (43%). Importantly, in eight of these patients, 3 had abnormal findings in only one of the two modalities, while the other five had positive findings on both images, but with different distributions. These findings are important as they indicate the potential power of orthogonal data from multimodality imaging to increase test sensitivity. This kind of data has been replicated in numerous case studies using hybrid or same day PET-CMR [44–49]. Together these data indicate that the hybrid approach yields superior diagnostic information than either modality alone.

### Amyloid

**Fig. 2:**
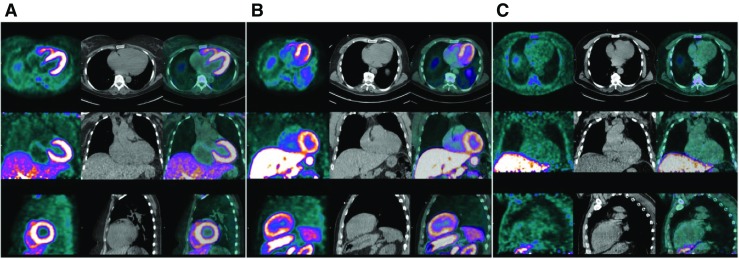
^18^F–florbetaben PET (left column in each panel), low-dose CT (middle column), and PET/CT images (right column) of representative AL patient (A), ATTR patient (B), and hypertensive control (C). ^18^F–florbetaben myocardial uptake is seen in patients with both AL and ATTR amyloidosis. W. Phillip Law et al. [50] J Nucl Med 2016;57:1733–1739

Cardiac amyloid deposits can be imaged using PET radiotracers, which target the amyloid β-pleated-sheet structure, for example ^18^F–florbetaben PET (Figs. 2, 50] CMR biomarkers of cardiac amyloidosis are of proven prognostic value [51]. However, CMR is unable to differentiate between the two main forms of amyloid: acquired monoclonal immunoglobulin light-chain (AL) and transthyretin-related (familial and wild-type/senile) amyloid (ATTR). This information has important prognostic and therapeutic implications. Nuclear imaging with SPECT bone tracers has been shown as one possible method to differentiate amyloid subtypes [52, 53]. Trivieri et al. [54] used hybrid PET-CMR and the PET bone tracer ^18^F–sodium fluoride, with the aim of differentiation of ATTR and AL forms within a single, low-radiation scan. Fourteen subjects were prospectively recruited, comprising 4 patients with biopsy-proven ATTR, 3 with biopsy-proven AL amyloid, and 7 control subjects without clinical suspicion of amyloid. The authors performed image analysis on fused, co-registered PET and MR LGE images, allowing PET activity to be measured within the LV myocardium, and importantly demonstrating specific areas of amyloid deposition that were visualized on LGE. The amyloid burden as assessed by T1 mapping was similar in patients with AL and ATTR amyloid, however patchy areas of increased ^18^F–sodium fluoride uptake were observed in the myocardium of patients with ATTR but not the other two groups. This study differs from some of the other approaches described in this review, in that truly independent (and complementary) data is derived from both modalities, rather than images which essentially describe the same pathology in different image space (e.g. ^18^F–FDG-PET and T1/T2 mapping for inflammation).

Novel PET radiotracers may have potential to further refine ATTR amyloid diagnosis. Amongst patients with ATTR V30 M (the most common Transthyretin [TTR] mutation in Europe) amyloidosis, two main phenotypes are observed: one characterized by late onset (>50 years) with both cardiac and neurological manifestations and another with an early onset of disease, with mainly neuropathic manifestations. These phenotypes have been associated with differences in amyloid fibril composition – type A (full length TTR and fragments) and type B (full length TTR) [55]. Pilebro et al. investigated the role of ^11^C–Pittsburgh compound B (PIB) to differentiate these subtypes of ATTR, and showed that global LV ^11^C–PIB was higher in patients with Type B V30 M ATTR [56].

### Myocarditis

**Fig. 3:**
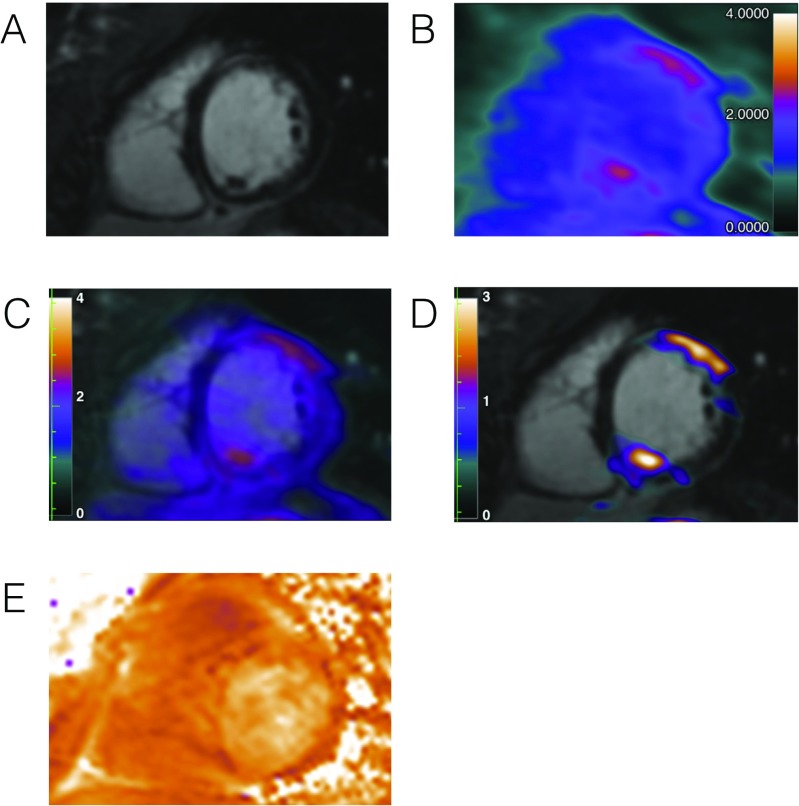
Patient With Acute Myocarditis. Patient (25-year-old female) hospitalized for supraventricular tachycardia with history of recent viral infection, and inconclusive myocardial biopsy. **(A)** LGE-CMR image obtained in short-axis view shows linear mid-wall and subepicardial LGE of the anterior wall and inferoseptum. (B) FDG PET imaging performed 90 min after injection of 370 MBq FDG following dietary restrictions to suppress myocardial tracer uptake demonstrated FDG uptake that matched LGE. **(C-D)** Fused FDG-PET/MR images demonstrated increased activity exactly corresponding to the pattern of injury on CMR (maximum standardized uptake value of LGE territory/blood pool uptake ratio = 2.0). **(E)** In the same view, CMR T_2_ mapping was unable to clearly differentiate regions of increased myocardial inflammation. This example illustrates the exact co-localization of the PET FDG signal with the pattern of injury on LGE, facilitating the diagnosis of active myocarditis. Figure adapted from Abgral et al. [47] JACC CV Imaging 2016

Acute myocarditis is an important cause of death in children and young adults and is traditionally assumed to account for many cases of dilated cardiomyopathy previously classified as idiopathic. The role of CMR (especially T1 and T2 mapping) is now established in the diagnosis of acute and chronic myocarditis [57, 58]. However, CMR fails to identify a significant number of patients with biopsy proven myocarditis. The addition of nuclear imaging to CMR may therefore improve sensitivity, Fig. 3.

Nensa et al. investigated the feasibility of PET-CMR for the investigation of suspected myocarditis [59]. The authors showed good agreement between myocardial FDG and LGE/T2 hyper-intensity. Overall 24/65 patients had abnormalities on either PET or CMR imaging consistent with myocarditis: 17 patients (71%) showed concordant CMR and PET abnormalities, 6 patients (25%) had pathologic CMR findings in the absence of pathologic FDG uptake and 1 patient (4%) demonstrated pathologic FDG uptake in the absence of pathologic CMR findings. These data point to several important problems for PET-CMR: 1) in 8/65 patients (12%), the inhibition of physiological myocardial glucose uptake failed, rendering the nuclear imaging unusable, and 2) adding PET imaging provided only a minimal increase in the sensitivity above CMR imaging alone (also assuming this was not a false positive). On this basis, the case has not been clearly made for routine use of PET-CMR in the assessment of myocarditis.

### Anderson fabry disease

Anderson Fabry disease (AFD) is an x-linked lysosomal storage disorder. It is caused by deficient activity of the lysosomal enzyme alpha-galactosidase A, resulting in the accumulation of globotriaosylceramide in lysosomes in multiple cell types throughout the body [60]. Heart failure is the most common first cardiac event in AFD patients [61], but may be preventable with enzyme replacement therapy; [62–64] early diagnosis of cardiac involvement is therefore important. Nappi et al. [65] used PET-CMR hybrid imaging to assess cardiac involvement a cohort of 13 asymptomatic AFD patients. There were three components to the imaging protocol: LGE imaging (fibrosis), short inversion time inversion recovery [STIR] imaging (edema), and ^18^F–FDG PET (myocardial metabolism). 6/13 patients exhibited focal LGE indicating intra-myocardial fibrosis; four of these patients also had positive STIR imaging suggesting myocardial edema. All 4 patients with LGE and positive STIR showed focal FDG uptake in the corresponding myocardial segments indicating inflammation (these patients also had elevated cardiac troponin). An important area requiring further exploration is the significance of heterogenous FDG uptake in a group of patients without other CMR abnormalities - Does this finding represent myocardial inflammation indicating early (pre-clinical) cardiac involvement? Alternatively, could enzyme replacement therapy itself result in inflammation secondary to globotriaosylceramide clearance? This novel data supports the role of inflammation in the pathogenesis of cardiac involvement in AFD, and demonstrates the possibility for dissecting disease stages using multimodality PET-CMR.

## Potential new developments

### Sympathetic nervous system imaging

Whilst PET-CMR literature remains in its infancy, it is possible to identify some areas of future development applicable to heart failure. Sympathetic nervous system imaging The ADMIRE-HF (AdreView Myocardial Imaging for Risk Evaluation in Heart Failure) study prospectively evaluated iodine-123 meta-iodobenzylguanidine (^123^I–mIBG) imaging for identifying cardiac events in patients with symptomatic heart failure. Increased myocardial sympathetic activity is a prominent feature of heart failure and is associated with reduced neuronal norepinephrine (NE) uptake due to post-transcriptional down-regulation of the cardiac NE transporter, which can be assessed by ^123^I–mIBG imaging. The ADMIRE-HF trial showed that mIBG data provides complementary prognostic information to existing heart failure biomarkers (BNP and LVEF) [66]. The role of the sympathetic nervous system can also be approached using PET tracers such as ^11^C meta-hydroxyephedrine (^11^C–HED) [67]. Data from ^123^I–mIBG and ^11^C–HED in heart failure have been shown to be analogous, with ^11^C–HED having superior capacity to identify regional abnormalities [68]. The PAREPET trial (Prediction of ARrhythmic Events with Positron Emission Tomography) investigated sympathetic denervation assessed using ^11^C–HED PET in ischemic cardiomyopathy. The study showed that ^11^C–HED PET predicted cause-specific mortality from sudden cardiac arrest independently of LVEF and infarct volume. These data may provide information useful for the identification of patients most likely to benefit from an ICD [69]. The potential of multiparametric assessment of ischemic cardiomyopathy investigated by De Haan et al. [70] used serial PET and CMR with ^11^C–HED (sympathetic innervation) and ^15^O–water (myocardial perfusion) to study the relationship between, scar, perfusion and innervation. The authors showed that areas of denervated, residual viable myocardium in ischemic cardiomyopathy were related to the heterogenic scar zone as assessed with LGE CMR. A novel ^18^F–labeled ligand for the norepinephrine transporter (N-[3-bromo-4-(3-^18^F-fluoro-propoxy)-benzyl]-guanidine [LMI1195]) is in clinical development for mapping cardiac nerve terminals in vivo using PET. First in man studies, have shown that LMI1195 is well tolerated and yields a radiation dose comparable to that of other commonly used PET radiopharmaceuticals [71]. The clinical and imaging significance of the autonomic nervous system for heart failure continues to develop [72].

### Myocardial metabolism

The failing heart undergoes significant, complex changes in energy utilization. In addition to Glucose imaging with FDG-PET, it is also possible to assess myocardial fatty acid utilization using novel metabolic tracers such as ^18^F–Fluoro-6-Thia-Heptadecanoic acid (FTHA). Taylor et al. [73] demonstrated the feasibility of this approach, demonstrating a reciprocal relationship between myocardial fatty acid and glucose uptake rates in heart failure; the former were higher than expected for the normal heart, whereas myocardial glucose uptake rates were lower. Metabolic assessment using MRI approaches are also becoming feasible, through the use of hyperpolarized carbon-13 imaging of pyruvate metabolism. In a preclinical model of porcine cardiomyopathy, Schroeder et al. [74] successfully demonstrated impaired pyruvate and fat oxidation at the onset of cardiomyopathy. However, whether myocardial metabolism represents a useful biomarker for heart failure remains under uncertain.

### Apoptosis

Apoptosis is considered an important pathophysiological element to the development of ventricular dysfunction in heart failure. Several molecular markers of programmed cell death exist, which are attractive targets for molecular imaging – for example phosphatidylserine (PS), which is translocated from the inner plasma layer to the cell surface under conditions of cell stress and physiologically serves as a marker to macrophages to remove apoptotic cells [75]. Several ligands with high specificity for PS exist, including Annexin V, which has been studied in heart failure using technetium SPECT, although experimental PET radiotracers also exist [76, 77]. Targeted magnetic nanoparticles for use in MRI, have also been used to image apoptosis in experimental animal models [78].

## Remodeling imaging: angiogenesis and fibrosis

Matrix metalloproteinases (MMPs) are a family of zinc-dependent, secreted or transmembrane enzymes that degrade components extracellular matrix (ECM). Angiogenesis, the process of forming new blood vessels, requires degradation of the vascular basement membrane and remodeling of the ECM in order to facilitate endothelial cell migration into the surrounding tissue. The relative contribution of these processes in response to myocardial injury is thought to be an important determinant of myocardial remodeling. Atrial fibrillation (AF) is a common arrhythmic complication in patients with heart failure. In patients with end-stage HF, left atrial and ventricular MMP-9 and collagen levels were higher in AF compared to patients with no AF [79]. Serological markers of collagen turnover have been demonstrated in patients with persistent AF, including elevated levels of TIMP-1 [80]. MMP radiotracers are under active investigation, but remain experimental, but could provide useful information on including arrhythmia risk in this population [81]. A novel α_v_β_3_ integrin-selective PET radiotracer, ^18^F–fluciclatide has been used to identify myocardial segments displaying functional recovery following acute myocardial infarction [82]. Imaging molecular targets such as α_v_β_3_ integrin (angiogenesis) or MMP activity may provide novel information about the myocardial remodeling process in patients with HF.

## Technical considerations

Several important technical considerations should briefly be mentioned. The contraindications for CMR imaging (e.g. pacemakers, CRT and ICD devices) and arrhythmia imaging challenges also apply to PET-CMR. These characteristics are prevalent in a heart failure population; therefore, general applicability is restricted.

Integration of PET and MRI hardware has been technically challenging, as conventional PET detectors are incompatible with the strong magnetic field intrinsic to MRI systems. Vendors have typically opted for either avalanche photodiodes or silicon photon multipliers that are insensitive to magnetic fields, and therefore can be integrated into hybrid units, Table 1.

**Table 1:** Examples of commercially available PET-MRI systems and their technical characteristics

Vendor	System Name	Characteristics	Technical Elements
Siemens	Biograph mMR	True Hybrid PET-MRI (single gantry)	3 T MRI Avalanche photodiode (APD) detectors with Lutetium oxyorthosilicate scintillator
GE	SIGNA™ PET/MR	True Hybrid PET-MRI (single gantry)	3 T MRI Silicon photomultiplier tube (SiPM) with Lutetium based scintillator.
Philips	Ingenuity TF PET/MR	Sequential PET and MRI (separate gantries, single room)	3 T MRI Photomultiplier tube (magnetically shielded) with Lutetium-yttrium oxyorthosilicate crystal scintillator

**Fig. 4:**
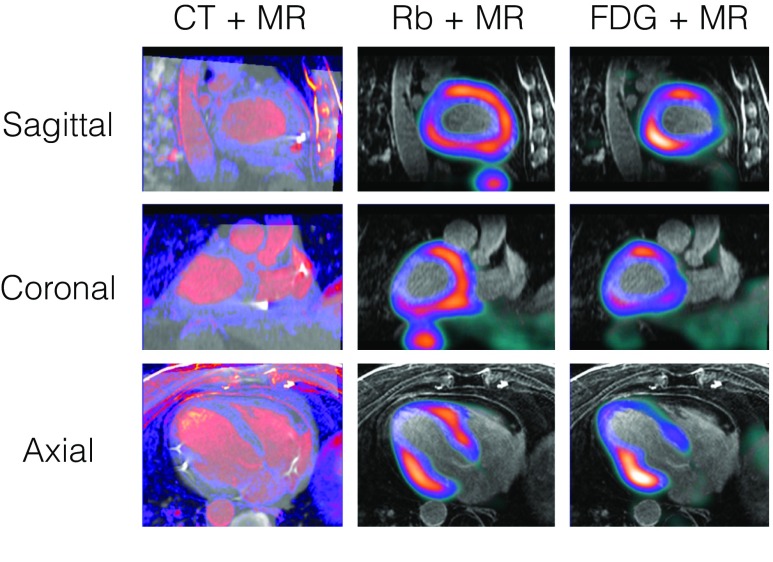
Image fusion of separately acquired PET/CT and CMR images in a patient with cardiac sarcoidosis. CT images from a hybrid PET-CT scanner were used to fuse the PET images (color) with MR images (B&W). Shown are fused CT and CMR images (left), fused ^82^Rb perfusion PET and CMR (middle), and fused ^18^FDG inflammation PET and CMR (right) studies. Image fusion and figure provided courtesy of Mary Germino, PhD, Yale University

One needs to also consider the true advantage of performing PET and MR imaging on a hybrid imaging system versus registering PET/CT images with MR images acquired on separate more conventional imaging systems, which might be operated independently with more clinical efficiency and at lower cost. Our group and others have demonstrated the feasibility of using the CT images from a hybrid PET/CT to accurately register separately acquired PET and MR images (Fig. 4).

PET-CMR infrastructure is incompatible with rubidium-82 generators, the most commonly utilized radiotracer for MPI. Alternative MPI radiotracers require an onsite cyclotron, thus limiting the accessibility of this technique. As an alternative for CAD assessment in such a situation, CMR MPI can be used.

**Fig. 5:**
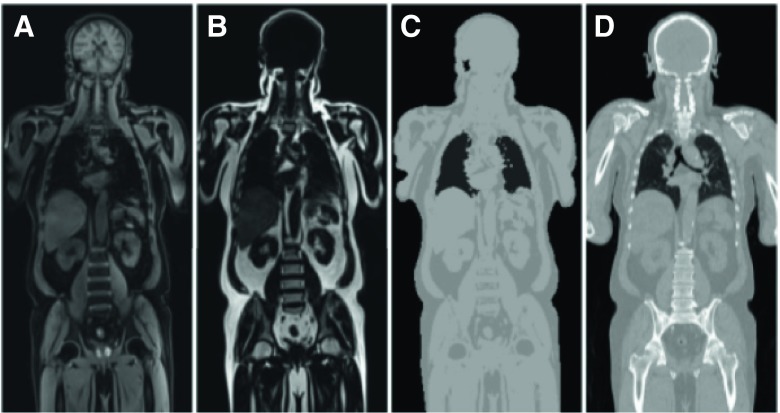
Dixon MRI-based attenuation correction used for PET-MRI. Shown are the Dixon water (A) and fat (B) images. C. MRI-based attenuation map was generated by combining water and fat images. D. CT of same patient. Adapted from Yoo et al. [84] (open access)

PET data needs to be attenuation corrected (AC) during reconstruction in order validly quantify tracer activity distribution. In PET-CT hybrid imaging, the CT image provides a spatial representation of the patient tissues and hardware components, which can be used to calculate attenuation maps. PET-MRI systems cannot measure linear attenuation directly; therefore, AC here needs to be performed differently. AC based on MRI data is typically performed using a Dixon-based MR acquisition, which defines air, fat, muscle, and lungs (Fig. 5). Using this approach, bone is classified as soft tissue, and thus signal attenuation of the bone is likely underestimated [83]. The obvious benefit is a reduced radiation dose using PET-MRI.

One important advantage of PET-CMR over PET-CT is the potential for motion correction. In PET-CT, the CT data is acquired once, and used for attenuation correction and anatomical localization. With hybrid PET-MRI, data may be acquired throughout the examination allowing for cardiac motion correction. Petibon et al. investigated the effects of motion correction and gating on the quantification of myocardial blood flow. Motion correction and gating, respectively decreased mean apparent wall thickness by 15.1% and 14.4% and increased MBR by 20.3% and 13.6% compared to ungated images.

An overview of the relative advantages and disadvantages of hybrid PET-MRI is given in Table 2.

**Table 2:** Relative advantages (**↑**), disadvantages (**↓**), no change (−) in value of imaging modalities in heart failure

	MRI	PET	PET-CT	PET-MRI	Advantage PET-MRI
Radiation	**↑↑↑**	**↓**	**↓↓**	**↓**	**↑↑**
Function	**↑↑↑**	**↑**	**↑**	**↑↑↑**	**-**
Tissue Characteristics	**↑↑↑**	**↑**	**↑**	**↑↑↑**	**-**
Perfusion Imaging	**↑↑**	**↑↑↑**	**↑↑↑**	**↑↑**	**↓**
Molecular Imaging	**↑**	**↑↑↑**	**↑↑↑**	**↑↑↑**	**↑↑**
ICD/PPM compatibility	**↓**	**↑**	**↑**	**↓**	**-**
Arrhythmia	**↓**	**↑**	**↑**	**↓**	**-**
PET Attenuation Correction	N/A	N/A	**↑↑↑**	**↑**	**↓**
PET Motion Correction	N/A	**↑**	**↑**	**↑↑↑**	**↑↑**

## Conclusion

Heart failure applications of PET-CMR are currently feasible. Unfortunately, the field remains in its infancy, and investigators are still addressing the challenges associated with the complexities of the technology. The future of this technology rests on the ability to achieve synergistic imaging – where non-redundant data, is combined to produce information greater than the sum of the parts. The challenge facing the nuclear component of this hybrid is the encroachment of radiation-free CMR techniques into its traditional territory (CMR perfusion imaging, T1/T2 mapping of inflammation). Using PET to target specific molecular processes, currently inaccessible to CMR is vital to the success of this powerful technology. Comparative effectiveness research is required to justify the need for concurrent PET-CMR imaging relative to PET/CT or MR imaging performed separately. These imaging modalities are already adequate for some clinical assessments and are undoubtedly less expensive and logistically less complex.

Is PET-CMR in heart failure ready for prime time? Not yet, but there are sufficient clues in the data presented here that synergistic multi-modality imaging may be on the horizon.

## References

[1] Cook C, Cole G, Asaria P, Jabbour R, Francis DP (2014). The annual global economic burden of heart failure. Int J Cardiol.

[2] Bailey DL, Pichler BJ, Guckel B, Barthel H, Beer AJ, Botnar R, Gillies R, Goh V, Gotthardt M, Hicks RJ, Lanzenberger R, la Fougere C, Lentschig M, Nekolla SG, Niederdraenk T, Nikolaou K, Nuyts J, Olego D, Riklund KA, Signore A, Schafers M, Sossi V, Suminski M, Veit-Haibach P, Umutlu L, Wissmeyer M, Beyer T (2016). Combined PET/MRI: from status quo to status go. Summary report of the fifth international workshop on PET/MR imaging; February 15-19, 2016; Tubingen, Germany. Mol Imaging Biol.

[3] Bart BA, Shaw LK, McCants CB, Fortin DF, Lee KL, Califf RM, O'Connor CM (1997). Clinical determinants of mortality in patients with angiographically diagnosed ischemic or nonischemic cardiomyopathy. J Am Coll Cardiol.

[4] Petibon Y, Guehl NJ, Reese TG, Ebrahimi B, Normandin MD, Shoup TM, Alpert NM, El Fakhri G, Ouyang J (2016). Impact of motion and partial volume effects correction on PET myocardial perfusion imaging using simultaneous PET-MR. Phys Med Biol.

[5] Dilsizian V, Bacharach SL, Beanlands RS, Bergmann SR, Delbeke D, Gropler RJ (2009). ASNC imaging guidelines for nuclear cardiology procedures: PET myocardial perfusion and metabolism clinical imaging. J Nucl Cardiol.

[6] Sinusas AJ (2010). Molecular imaging in nuclear cardiology: translating research concepts into clinical applications. Q J Nucl Med Mol Imaging.

[7] Schindler TH, Schelbert HR, Quercioli A, Dilsizian V (2010). Cardiac PET imaging for the detection and monitoring of coronary artery disease and microvascular health. J Am Coll Cardiol Img.

[8] Uren NG, Melin JA, De Bruyne B, Wijns W, Baudhuin T, Camici PG (1994). Relation between myocardial blood flow and the severity of coronary-artery stenosis. New Engl J Med.

[9] Ziadi MC, Williams KA, Guo A, Chow BJ, Renaud JM, Ruddy TD, Sarveswaran N, Tee RE, Beanlands RS (2011). Impaired myocardial flow reserve on rubidium-82 positron emission tomography imaging predicts adverse outcomes in patients assessed for myocardial ischemia. J Am Coll Cardiol.

[10] Herzog BA, Husmann L, Valenta I, Gaemperli O, Siegrist PT, Tay FM, Burkhard N, Wyss CA, Kaufmann PA (2009). Long-term prognostic value of 13 N-ammonia myocardial perfusion positron emission tomography: added value of coronary flow reserve. J Am Coll Cardiol.

[11] Steeden JA, Knight DS, Bali S, Atkinson D, Taylor AM, Muthurangu V (2014). Self-navigated tissue phase mapping using a golden-angle spiral acquisition-proof of concept in patients with pulmonary hypertension. Magnetic resonance in medicine : official journal of the Society of Magnetic Resonance in Medicine / Society of Magnetic Resonance in Medicine.

[12] Budge LP, Helms AS, Salerno M, Kramer CM, Epstein FH, Bilchick KC (2012). MR cine DENSE dyssynchrony parameters for the evaluation of heart failure: comparison with myocardial tissue tagging. JACC Cardiovasc Imaging.

[13] Bellenger NG, Davies LC, Francis JM, Coats AJ, Pennell DJ (2000). Reduction in sample size for studies of remodeling in heart failure by the use of cardiovascular magnetic resonance. J Cardiovasc Magn Reson.

[14] Morton G, Chiribiri A, Ishida M, Hussain ST, Schuster A, Indermuehle A, Perera D, Knuuti J, Baker S, Hedström E, Schleyer P, O'Doherty M, Barrington S, Nagel E (2012). Quantification of absolute myocardial perfusion in patients with coronary artery disease: comparison between cardiovascular magnetic resonance and positron emission tomography. J Am Coll Cardiol.

[15] Christian TF, Rettmann DW, Aletras AH, Liao SL, Taylor JL, Balaban RS, Arai AE (2004). Absolute myocardial perfusion in canines measured by using dual-bolus first-pass MR imaging 1. Radiology.

[16] Levy D, Kenchaiah S, Larson MG, Benjamin EJ, Kupka MJ, Ho KK, Murabito JM, Vasan RS (2002). Long-term trends in the incidence of and survival with heart failure. New Engl J Med.

[17] Taylor AL, Ziesche S, Yancy C, Carson P, D'Agostino R, Ferdinand K, Taylor M, Adams K, Sabolinski M, Worcel M (2004). Combination of isosorbide dinitrate and hydralazine in blacks with heart failure. New Engl J Med.

[18] Velazquez EJ, Lee KL, Deja MA, Jain A, Sopko G, Marchenko A, Ali IS, Pohost G, Gradinac S, Abraham WT (2011). Coronary-artery bypass surgery in patients with left ventricular dysfunction. New Engl J Med.

[19] Doshi D, Ben-Yehuda O, Bonafede M, Josephy N, Karmpaliotis D, Parikh MA, Moses JW, Stone GW, Leon MB, Schwartz A, Kirtane AJ (2016). Underutilization of coronary artery disease testing among patients hospitalized with new-onset heart failure. J Am Coll Cardiol.

[20] Parker MW, Iskandar A, Limone B, Perugini A, Kim H, Jones C, Calamari B, Coleman CI, Heller GV (2012). Diagnostic accuracy of cardiac positron emission tomography versus single photon emission computed tomography for coronary artery disease: a bivariate meta-analysis. Circulation: Cardiovascular Imaging.

[21] Hachamovitch R, Hayes SW, Friedman JD, Cohen I, Berman DS (2003). Comparison of the short-term survival benefit associated with revascularization compared with medical therapy in patients with no prior coronary artery disease undergoing stress myocardial perfusion single photon emission computed tomography. Circulation.

[22] Lau J, Laforest R, Zheng J, Lesniak D, Priatna A, Gropler R, Woodard P (2014). 13N-ammonia PET/MR myocardial stress perfusion imaging early experience. J Nucl Med.

[23] Durrani A, Lau J, Laforest R, Zheng J, Lesniak D, Priatna A, Gropler R, Woodard P (2016) 13N–ammonia PET/MR myocardial stress perfusion imaging early experience. In: Radiological Society of North America 2015 Scientific Assembly and Annual Meeting. http://archive.rsna.org/2015/15012463.html

[24] Lipinski MJ, McVey CM, Berger JS, Kramer CM, Salerno M (2013). Prognostic value of stress cardiac magnetic resonance imaging in patients with known or suspected coronary artery disease: a systematic review and meta-analysis. J Am Coll Cardiol.

[25] Gargiulo P, Dellegrottaglie S, Bruzzese D, Savarese G, Scala O, Ruggiero D, D'Amore C, Paolillo S, Agostoni P, Bossone E, Soricelli A, Cuocolo A, Trimarco B, Perrone Filardi P (2013). The prognostic value of normal stress cardiac magnetic resonance in patients with known or suspected coronary artery disease: a meta-analysis. Circ Cardiovasc Imaging.

[26] Allman KC, Shaw LJ, Hachamovitch R, Udelson JE (2002). Myocardial viability testing and impact of revascularization on prognosis in patients with coronary artery disease and left ventricular dysfunction: a meta-analysis. J Am Coll Cardiol.

[27] Beanlands RSB, Nichol G, Huszti E, Humen D, Racine N, Freeman M, Gulenchyn KY, Garrard L, deKemp R, Guo A, Ruddy TD, Benard F, Lamy A, Iwanochko RM (2007). F-18-fluorodeoxyglucose positron emission tomography imaging-assisted Management of Patients with Severe Left Ventricular Dysfunction and Suspected Coronary Disease. A randomized, controlled trial (PARR-2). J Am Coll Cardiol.

[28] D'Egidio G, Nichol G, Williams KA, Guo A, Garrard L, deKemp R, Ruddy TD, DaSilva J, Humen D, Gulenchyn KY, Freeman M, Racine N, Benard F, Hendry P, Beanlands RSB (2009). Increasing benefit from revascularization is associated with increasing amounts of myocardial hibernation: a substudy of the PARR-2 trial. J Am Coll Cardiol Img.

[29] Bonow RO, Maurer G, Lee KL, Holly TA, Binkley PF, Desvigne-Nickens P, Drozdz J, Farsky PS, Feldman AM, Doenst T (2011). Myocardial viability and survival in ischemic left ventricular dysfunction. New Engl J Med.

[30] Srichai MB, Jaber WA (2013). Viability by MRI or PET would have changed the results of the STICH trial. Prog Cardiovasc Dis.

[31] Ling LF, Marwick TH, Flores DR, Jaber WA, Brunken RC, Cerqueira MD, Hachamovitch R (2013). Identification of therapeutic benefit from revascularization in patients with left ventricular systolic dysfunction inducible ischemia versus hibernating myocardium. Circulation: Cardiovascular Imaging.

[32] Kim RJ, Wu E, Rafael A, Chen E-L, Parker MA, Simonetti O, Klocke FJ, Bonow RO, Judd RM (2000). The use of contrast-enhanced magnetic resonance imaging to identify reversible myocardial dysfunction. New Engl J Med.

[33] Klein C, Nekolla SG, Bengel FM, Momose M, Sammer A, Haas F, Schnackenburg B, Delius W, Mudra H, Wolfram D (2002). Assessment of myocardial viability with contrast-enhanced magnetic resonance imaging comparison with positron emission tomography. Circulation.

[34] Beanlands RS, Ruddy TD, Iwanochko RM, Coates G, Freeman M, Nahmias C, Hendry P, Burns RJ, Lamy A, Mickleborough L (2002). Positron emission tomography and recovery following revascularization (PARR-1): the importance of scar and the development of a prediction rule for the degree of recovery of left ventricular function. J Am Coll Cardiol.

[35] Romero J, Xue X, Gonzalez W, Garcia MJ (2012). CMR imaging assessing viability in patients with chronic ventricular dysfunction due to coronary artery disease: a meta-analysis of prospective trials. J Am Coll Cardiol Img.

[36] Bulluck H, White SK, Frohlich GM, Casson SG, O'Meara C, Newton A, Nicholas J, Weale P, Wan SM, Sirker A, Moon JC, Yellon DM, Groves A, Menezes L, Hausenloy DJ (2016). Quantifying the area at risk in reperfused ST-segment-elevation myocardial infarction patients using hybrid cardiac positron emission tomography-magnetic resonance imaging. Circ Cardiovasc Imaging.

[37] Rischpler C, Langwieser N, Souvatzoglou M, Batrice A, van Marwick S, Snajberk J, Ibrahim T, Laugwitz KL, Nekolla SG, Schwaiger M (2015). PET/MRI early after myocardial infarction: evaluation of viability with late gadolinium enhancement transmurality vs. 18F-FDG uptake. Eur Heart J Cardiovasc Imaging.

[38] Robson PM, Dweck MR, Trivieri MG, Abgral R, Karakatsanis NA, Contreras J, Gidwani U, Narula JP, Fuster V, Kovacic JC, Fayad ZA (2017). Coronary artery PET/MR imaging. Feasibility, Limitations, and Solutions.

[39] Hamzeh N, Steckman DA, Sauer WH, Judson MA (2015). Pathophysiology and clinical management of cardiac sarcoidosis. Nat Rev Cardiol.

[40] Smedema JP, Snoep G, van Kroonenburgh MP, van Geuns RJ, Dassen WR, Gorgels AP, Crijns HJ (2005). Cardiac involvement in patients with pulmonary sarcoidosis assessed at two university medical centers in the Netherlands. Chest.

[41] Patel MR, Cawley PJ, Heitner JF, Klem I, Parker MA, Jaroudi WA, Meine TJ, White JB, Elliott MD, Kim HW, Judd RM, Kim RJ (2009). Detection of myocardial damage in patients with sarcoidosis. Circulation.

[42] Ishimaru S, Tsujino I, Takei T, Tsukamoto E, Sakaue S, Kamigaki M, Ito N, Ohira H, Ikeda D, Tamaki N, Nishimura M (2005). Focal uptake on 18F-fluoro-2-deoxyglucose positron emission tomography images indicates cardiac involvement of sarcoidosis. Eur Heart J.

[43] Ohira H, Tsujino I, Ishimaru S, Oyama N, Takei T, Tsukamoto E, Miura M, Sakaue S, Tamaki N, Nishimura M (2008). Myocardial imaging with 18F-fluoro-2-deoxyglucose positron emission tomography and magnetic resonance imaging in sarcoidosis. Eur J Nucl Med Mol Imaging.

[44] O'Meara C, Menezes LJ, White SK, Wicks E, Elliott P (2013). Inital experience of imaging cardiac sarcoidosis using hybrid PET-MR - a technologist's case study. J Cardiovasc Magn Reson.

[45] Schneider S, Batrice A, Rischpler C, Eiber M, Ibrahim T, Nekolla SG (2014). Utility of multimodal cardiac imaging with PET/MRI in cardiac sarcoidosis: implications for diagnosis, monitoring and treatment. Eur Heart J.

[46] Wada K, Niitsuma T, Yamaki T, Masuda A, Ito H, Kubo H, Hara T, Takenoshita S, Takeishi Y (2016). Simultaneous cardiac imaging to detect inflammation and scar tissue with (18)F-fluorodeoxyglucose PET/MRI in cardiac sarcoidosis. J Nucl Cardiol.

[47] Abgral R, Dweck MR, Trivieri MG, Robson PM, Karakatsanis N, Mani V, Padilla M, Miller M, Lala A, Sanz J, Narula J, Fuster V, Contreras J, Kovacic JC, Fayad ZA (2016). Clinical utility of combined FDG-PET/MR to assess myocardial disease. J Am Coll Cardiol Img.

[48] Coulden R, Jones H, Sonnex E, Das I, Abele J (2014). Cardiac MRI and FDG-PET in the diagnosis of cardiac sarcoidosis. J Cardiovasc Magn Reson.

[49] Galati G, Leone O, Rapezzi C (2014). The difficult diagnosis of isolated cardiac sarcoidosis: usefulness of an integrated MRI and PET approach. Heart.

[50] Law WP, Wang WY, Moore PT, Mollee PN, Ng AC (2016). Cardiac amyloid imaging with 18F-florbetaben PET: a pilot study. Journal of nuclear medicine : official publication, Society of Nuclear Medicine.

[51] Fontana M, Pica S, Reant P, Abdel-Gadir A, Treibel TA, Banypersad SM, Maestrini V, Barcella W, Rosmini S, Bulluck H (2015). Prognostic value of late gadolinium enhancement cardiovascular magnetic resonance in cardiac amyloidosis. Circulation:CIRCULATIONAHA..

[52] Gillmore JD, Maurer MS, Falk RH, Merlini G, Damy T, Dispenzieri A, Wechalekar AD, Berk JL, Quarta CC, Grogan M (2016). Non-biopsy diagnosis of cardiac transthyretin amyloidosis. Circulation:CIRCULATIONAHA.

[53] Rapezzi C, Quarta CC, Guidalotti PL, Pettinato C, Fanti S, Leone O, Ferlini A, Longhi S, Lorenzini M, Reggiani LB, Gagliardi C, Gallo P, Villani C, Salvi F (2011). Role of 99mTc-DPD scintigraphy in diagnosis and prognosis of hereditary transthyretin-related cardiac amyloidosis. J Am Coll Cardiol Img.

[54] Trivieri MG, Dweck MR, Abgral R, Robson PM, Karakatsanis NA, Lala A, Contreras J, Sahni G, Gopalan R, Gorevic P, Fuster V, Narula J, Fayad ZA (2016). 18F-sodium fluoride PET/MR for the assessment of cardiac amyloidosis. J Am Coll Cardiol.

[55] Ihse E, Ybo A, Suhr O, Lindqvist P, Backman C, Westermark P (2008). Amyloid fibril composition is related to the phenotype of hereditary transthyretin V30M amyloidosis. J Pathol.

[56] Pilebro B, Arvidsson S, Lindqvist P, Sundstrom T, Westermark P, Antoni G, Suhr O, Sorensen J (2016). Positron emission tomography (PET) utilizing Pittsburgh compound B (PIB) for detection of amyloid heart deposits in hereditary transthyretin amyloidosis (ATTR). J Nucl Cardiol.

[57] Lurz P, Eitel I, Adam J, Steiner J, Grothoff M, Desch S, Fuernau G, de Waha S, Sareban M, Luecke C (2012). Diagnostic performance of CMR imaging compared with EMB in patients with suspected myocarditis. J Am Coll Cardiol Img.

[58] Lurz P, Luecke C, Eitel I, Fohrenbach F, Frank C, Grothoff M, de Waha S, Rommel KP, Lurz JA, Klingel K, Kandolf R, Schuler G, Thiele H, Gutberlet M (2016). Comprehensive cardiac magnetic resonance imaging in patients with suspected myocarditis: the MyoRacer-trial. J Am Coll Cardiol.

[59] Nensa F, Poeppel TD, Krings P, Schlosser T (2014). Multiparametric assessment of myocarditis using simultaneous positron emission tomography/magnetic resonance imaging. Eur Heart J.

[60] Gambarin FI, Disabella E, Narula J, Diegoli M, Grasso M, Serio A, Favalli BM, Agozzino M, Tavazzi L, Fraser AG, Arbustini E (2010). When should cardiologists suspect Anderson-fabry disease?. Am J Cardiol.

[61] Patel MR, Cecchi F, Cizmarik M, Kantola I, Linhart A, Nicholls K, Strotmann J, Tallaj J, Tran TC, West ML, Beitner-Johnson D, Abiose A (2011). Cardiovascular events in patients with fabry disease natural history data from the fabry registry. J Am Coll Cardiol.

[62] Imbriaco M, Pisani A, Spinelli L, Cuocolo A, Messalli G, Capuano E, Marmo M, Liuzzi R, Visciano B, Cianciaruso B, Salvatore M (2009). Effects of enzyme-replacement therapy in patients with Anderson-fabry disease: a prospective long-term cardiac magnetic resonance imaging study. Heart.

[63] Thurberg BL, Fallon JT, Mitchell R, Aretz T, Gordon RE, O'Callaghan MW (2009). Cardiac microvascular pathology in fabry disease: evaluation of endomyocardial biopsies before and after enzyme replacement therapy. Circulation.

[64] Weidemann F, Niemann M, Breunig F, Herrmann S, Beer M, Stork S, Voelker W, Ertl G, Wanner C, Strotmann J (2009). Long-term effects of enzyme replacement therapy on fabry cardiomyopathy: evidence for a better outcome with early treatment. Circulation.

[65] Nappi C, Altiero M, Imbriaco M, Nicolai E, Giudice CA, Aiello M, Diomiaiuti CT, Pisani A, Spinelli L, Cuocolo A (2015). First experience of simultaneous PET/MRI for the early detection of cardiac involvement in patients with Anderson-fabry disease. Eur J Nucl Med Mol Imaging.

[66] Jacobson AF, Senior R, Cerqueira MD, Wong ND, Thomas GS, Lopez VA, Agostini D, Weiland F, Chandna H, Narula J (2010). Myocardial iodine-123 meta-iodobenzylguanidine imaging and cardiac events in heart failure: results of the prospective ADMIRE-HF (AdreView myocardial imaging for risk evaluation in heart failure) study. J Am Coll Cardiol.

[67] Hartmann F, Ziegler S, Nekolla S, Hadamitzky M, Seyfarth M, Richardt G, Schwaiger M (1999). Regional patterns of myocardial sympathetic denervation in dilated cardiomyopathy: an analysis using carbon-11 hydroxyephedrine and positron emission tomography. Heart.

[68] Matsunari I, Aoki H, Nomura Y, Takeda N, Chen W-P, Taki J, Nakajima K, Nekolla SG, Kinuya S, Kajinami K (2010). Iodine-123 Metaiodobenzylguanidine imaging and carbon-11 hydroxyephedrine positron emission tomography compared in patients with left ventricular DysfunctionClinical perspective. Circulation: Cardiovascular Imaging.

[69] Fallavollita JA, Heavey BM, Luisi AJ, Michalek SM, Baldwa S, Mashtare TL, Hutson AD, deKemp RA, Haka MS, Sajjad M, Cimato TR, Curtis AB, Cain ME, Canty JM (2014). Regional myocardial sympathetic denervation predicts the risk of sudden cardiac arrest in ischemic cardiomyopathy. J Am Coll Cardiol.

[70] de Haan S, Rijnierse MT, Harms HJ, Verberne HJ, Lammertsma AA, Huisman MC, Windhorst AD, van Rossum AC, Allaart CP, Knaapen P (2016). Myocardial denervation coincides with scar heterogeneity in ischemic cardiomyopathy: a PET and CMR study. J Nucl Cardiol.

[71] Sinusas AJ, Lazewatsky J, Brunetti J, Heller G, Srivastava A, Liu YH, Sparks R, Puretskiy A, Lin SF, Crane P, Carson RE, Lee LV (2014). Biodistribution and radiation dosimetry of LMI1195: first-in-human study of a novel 18F-labeled tracer for imaging myocardial innervation. Journal of nuclear medicine : official publication, Society of Nuclear Medicine.

[72] Thackeray JT, Bengel FM (2013). Assessment of cardiac autonomic neuronal function using PET imaging. J Nucl Cardiol.

[73] Taylor M, Wallhaus TR, Degrado TR, Russell DC, Stanko P, Nickles RJ, Stone CK (2001). An evaluation of myocardial fatty acid and glucose uptake using PET with [18F]fluoro-6-thia-heptadecanoic acid and [18F]FDG in patients with congestive heart failure. Journal of nuclear medicine : official publication, Society of Nuclear Medicine.

[74] Schroeder MA, Lau AZ, Chen AP, Gu Y, Nagendran J, Barry J, Hu X, Dyck JR, Tyler DJ, Clarke K (2013). Hyperpolarized 13C magnetic resonance reveals early-and late-onset changes to in vivo pyruvate metabolism in the failing heart. Eur J Heart Fail.

[75] Korngold EC, Jaffer FA, Weissleder R, Sosnovik DE (2008). Noninvasive imaging of apoptosis in cardiovascular disease. Heart Fail Rev.

[76] Kietselaer BL, Reutelingsperger CP, Boersma HH, Heidendal GA, Liem H, Crijns HJ, Narula J, Hofstra L (2007). Noninvasive detection of programmed cell loss with 99mTc-labeled annexin A5 in heart failure. J Nucl Med.

[77] Grierson JR, Yagle KJ, Eary JF, Tait JF, Gibson DF, Lewellen B, Link JM, Krohn KA (2004). Production of [F-18] fluoroannexin for imaging apoptosis with PET. Bioconjug Chem.

[78] Sosnovik DE, Schellenberger EA, Nahrendorf M, Novikov MS, Matsui T, Dai G, Reynolds F, Grazette L, Rosenzweig A, Weissleder R, Josephson L (2005). Magnetic resonance imaging of cardiomyocyte apoptosis with a novel magneto-optical nanoparticle. Magnetic resonance in medicine : official journal of the Society of Magnetic Resonance in Medicine / Society of Magnetic Resonance in Medicine.

[79] Mukherjee R, Herron AR, Lowry AS, Stroud RE, Stroud MR, Wharton JM, Ikonomidis JS, Crumbley AJ, Spinale FG, Gold MR (2006). Selective induction of matrix metalloproteinases and tissue inhibitor of metalloproteinases in atrial and ventricular myocardium in patients with atrial fibrillation. Am J Cardiol.

[80] Kallergis EM, Manios EG, Kanoupakis EM, Mavrakis HE, Arfanakis DA, Maliaraki NE, Lathourakis CE, Chlouverakis GI, Vardas PE (2008). Extracellular matrix alterations in patients with paroxysmal and persistent atrial fibrillation: biochemical assessment of collagen type-I turnover. J Am Coll Cardiol.

[81] Sahul ZH, Mukherjee R, Song J, McAteer J, Stroud RE, Dione DP, Staib L, Papademetris X, Dobrucki LW, Duncan JS, Spinale FG, Sinusas AJ (2011). Targeted imaging of the spatial and temporal variation of matrix metalloproteinase activity in a porcine model of postinfarct remodeling: relationship to myocardial dysfunction. Circ Cardiovasc Imaging.

[82] Jenkins WS, Vesey AT, Stirrat C, Connell M, Lucatelli C, Neale A, Moles C, Vickers A, Fletcher A, Pawade T, Wilson I, Rudd JH, van Beek EJ, Mirsadraee S, Dweck MR, Newby DE (2016). Cardiac alphaVbeta3 integrin expression following acute myocardial infarction in humans. Heart.

[83] Martinez-Moller A, Souvatzoglou M, Delso G, Bundschuh RA, Chefd'hotel C, Ziegler SI, Navab N, Schwaiger M, Nekolla SG (2009). Tissue classification as a potential approach for attenuation correction in whole-body PET/MRI: evaluation with PET/CT data. Journal of nuclear medicine : official publication, Society of Nuclear Medicine.

[84] Yoo HJ, Lee JS, Lee JM (2015). Integrated whole body MR/PET: where are We?. Korean J Radiol.

